# Differential effects of arsenic trioxide on chemosensitization in human hepatic tumor and stellate cell lines

**DOI:** 10.1186/1471-2407-12-402

**Published:** 2012-09-10

**Authors:** Fatima Rangwala, Kevin P Williams, Ginger R Smith, Zainab Thomas, Jennifer L Allensworth, H Kim Lyerly, Anna Mae Diehl, Michael A Morse, Gayathri R Devi

**Affiliations:** 1 Department of Medicine, 2606 Duke University Medical Center, Durham, NC, 27710, USA; 2 Biomanufacturing Research Institute and Technology Enterprise (BRITE), Department of Pharmaceutical Sciences, Durham, North Carolina Central University, Durham, NC, 27707, USA; 3 Department of Surgery, Duke University Medical Center, Durham, NC, 27710, USA; 4 Duke Cancer Institute, Duke University Medical Center, Durham, NC, 27710, USA

**Keywords:** Hepatocellular carcinoma, Arsenic trioxide, Sorafenib, 5-fluorouracil, Apoptosis, High throughput assay, Stroma

## Abstract

**Background:**

Crosstalk between malignant hepatocytes and the surrounding peritumoral stroma is a key modulator of hepatocarcinogenesis and therapeutic resistance. To examine the chemotherapy resistance of these two cellular compartments *in vitro*, we evaluated a well-established hepatic tumor cell line, HepG2, and an adult hepatic stellate cell line, LX2. The aim was to compare the chemosensitization potential of arsenic trioxide (ATO) in combination with sorafenib or fluorouracil (5-FU), in both hepatic tumor cells and stromal cells.

**Methods:**

Cytotoxicity of ATO, 5-FU, and sorafenib, alone and in combination against HepG2 cells and LX2 cells was measured by an automated high throughput cell-based proliferation assay. Changes in survival and apoptotic signaling pathways were analyzed by flow cytometry and western blot. Gene expression of the 5-FU metabolic enzyme, thymidylate synthase, was analyzed by real time PCR.

**Results:**

Both HepG2 and LX2 cell lines were susceptible to single agent sorafenib and ATO at 24 hr (ATO IC_50_: 5.3 μM in LX2; 32.7 μM in HepG2; Sorafenib IC_50_: 11.8 μM in LX2; 9.9 μM in HepG2). In contrast, 5-FU cytotoxicity required higher concentrations and prolonged (48–72 hr) drug exposure. Concurrent ATO and 5-FU treatment of HepG2 cells was synergistic, leading to increased cytotoxicity due in part to modulation of thymidylate synthase levels by ATO. Concurrent ATO and sorafenib treatment showed a trend towards increased HepG2 cytotoxicity, possibly due to a significant decrease in MAPK activation in comparison to treatment with ATO alone.

**Conclusions:**

ATO differentially sensitizes hepatic tumor cells and adult hepatic stellate cells to 5-FU and sorafenib. Given the importance of both of these cell types in hepatocarcinogenesis, these data have implications for the rational development of anti-cancer therapy combinations for the treatment of hepatocellular carcinoma (HCC).

## Background

Recent data has demonstrated that the crosstalk between malignant hepatocytes and non-parenchymal, stromal liver cells, or the hepatic stellate cells, is crucial to hepatocarcinogenesis [1]. *In vitro* hepatic stellate cells within the tumor microenvironment promote hepatocarcinoma cell growth, epithelial to mesenchymal transition (EMT), and tumor invasion [2-4]. *In vivo*, subcutaneous co-implantation of hepatic stellate cells and hepatocellular carcinoma cells into nude mice results in significantly increased tumor growth and tumor volumes in comparison to the implantation of hepatocellular carcinoma cells alone [5]. These studies highlight tumor-stroma interactions as a potential mediator of chemoresistance.

Arsenic trioxide (ATO), used for the treatment of relapsed acute promyelocytic leukemia, activates the caspase cascade and induces production of reactive oxygen species, resulting in apoptosis [6]. Multiple groups initially demonstrated that treatment of HCC cell lines with ATO inhibited cell growth and induced apoptosis in a concentration-dependent manner [7-9]. Furthermore, both in a rat model of diethaylnitrosamine-induced HCC and in murine HCC xenografts, ATO treatment significantly increased rates of apoptosis in tumor nodules in comparison to vehicle control [10,11]. Unfortunately, in a phase II clinical trial, single agent ATO did not show activity against advanced hepatocellular carcinoma (HCC) [12]. Possible explanations for the discrepant results between the preclinical data and the clinical data include HCC tumor heterogeneity both in humans and model systems. In addition, the preclinical model systems assessed cytotoxicity of ATO on the tumor compartment, but not on the supporting stromal compartment.

The pro-apoptotic effect of ATO can enhance the efficacy of other drugs given in combination as has been noted in other tumor types. In colorectal adenocarcinoma cells, ATO acts as a chemosensitizer to the pyrimidine antimetabolite 5-fluorouracil (5-FU) [13]. 5-FU, an inhibitor of thymidylate synthase (TS), was one of the first reported chemotherapeutic agents tested in the treatment of HCC with response rates of approximately 10% [14]. Capecitabine, an orally administered 5-FU prodrug, yielded response rates of 11% in patients with advanced HCC [15]. In addition to the benefit provided by this modest response rate, the fluoropyrimidines have a distinct advantage in the treatment of hepatobiliary malignancies in that they exhibit a favorable safety profile and do not require *a priori* dose reduction secondary to liver dysfunction [16,17].

Based on these data we were interested in comparing the chemosensitivity of the hepatic tumor compartment and the hepatic stromal compartment to single agent ATO, 5-FU and sorafenib. In the present study, the tumor compartment was modeled using a human, well-differentiated, epithelial hepatic tumor line, HepG2 [18], and the stromal compartment was modeled using the human hepatic stellate cell line, LX2 [19-21]. We hypothesized that the ATO/5-FU combination and the ATO/sorafenib combination would result in potentiation of apoptosis in the tumor and stromal cell line.

## Methods

### Cell culture

HepG2 (ATCC) cells and LX2 cells [21] were cultured as previously described [18,19]. Cells at 50% confluence in a 96-well plate (Corning Incorporated, Corning, NY, USA) were treated with increasing concentrations of arsenic trioxide (Cephalon, Philadelphia, PA), 5-fluorouracil (APP pharmaceuticals, Schaumburg, IL), or sorafenib (Bayer Healthcare Pharmaceuticals and Onyx Pharmaceuticals, San Francisco, CA) in serum-containing medium for the time periods indicated.

### Automated cell proliferation assay

HepG2 and LX2 cells were plated at 4000 and 3000 cells/well respectively in 96-well clear tissue culture plates (Costar, Corning Incorporated, Corning, NY) in 200 μl media and allowed to adhere overnight. Media was then removed from the cell plates and drug (200 μL) was transferred from intermediate plates to the cell plates using a Biomek NX workstation (Beckman Coulter Inc., Fullerton, CA) equipped with a 96-well head. For intermediate drug plates, using a Biomek 3000 workstation, drugs were serially diluted (eight concentrations) in media in 96-well 1.1 ml deep well plates (Axygen, VWR). For combination studies, the serial dilution was carried out at a 2-fold higher concentration and then a fixed concentration of a second drug added to all wells of the dose response intermediate plate. Each plate consisted of the following controls: column 1 contained media (no cells), columns 2 and 11 cells plus DMSO (final concentration 0.5%), and column 12 cells plus media. For combination experiments, column 11 would contain cells plus the second compound only. Cells were incubated with drugs for 24 hr, 48 hr, 72 hr and 96 hr and then cell proliferation assessed by an automated MTT assay adapted from our previous manual procedure [22]. Prior to preparation of cells for the MTT assay, all wells were viewed under an inverted scope to assess for the general number of attached cells in order to confirm correlation with assay values. Briefly, using the Biomek NX equipped with a 96-well head for all steps, MTT (40 μL) was added to the cell plates and incubated for 2 h. Media was then removed, DMSO (100 μL) added and the plates read at 550 nm in a SpectraMax Plus plate reader (Molecular Devices, Sunnyvale, CA, USA).

### Western blot analysis

Cells were treated as noted above and lysates were prepared. Western blot analysis was performed as previously described [22]. Membranes were incubated with primary antibodies against XIAP (1:2000; BD Bioscience), actin (1:1000; Santa Cruz Biotechnology), procaspase-9 (1:2000; NeoMarkers), and glyceraldehyde-3-phosphate dehydrogenase (GAPDH; 1:2000; Santa Cruz Biotechnology) for 1 h at room temperature. Membranes were incubated with primary antibodies against survivin (1:12000; R&D BioSystems), p-MAPK (1:1000; Cell Signaling), MAPK (1:1000; Cell Signaling), p-JNK (1:1000; Cell Signaling), and JNK (1:1000; Cell Signaling) overnight at 4°C.

### Drug synergism analysis

Analysis of drug synergism was performed using the Calcusyn software (Biosoft, Cambridge, UK), which uses the Chou-Talalay method [23] and generates summary statistics. The resultant combination index (CI) is a quantitative measurement of the relationship between two agents; a CI greater than 1 indicates antagonism, while a CI of one indicates an additive interaction and a CI less than one indicates synergism.

### Cell cycle analysis

Cells were treated with DMSO or arsenic trioxide (5 μM or 25 μM) for 24 hr and then stained with propidium iodide (PI) as previously reported [24]. At least 25,000 events were collected on a FACScalibur flow cytometer (Becton Dickinson) and analyzed using the Cellquest software (Becton Dickinson). Data presented represents n = 2 replicates.

### Quantitative real-time PCR for thymidylate synthase

HepG2 cells were seeded at 300,000 cells per well in clear, 6-well Costar plates and allowed to attach overnight. After treatment with vehicle (DMSO), 0.5 μM ATO, 5 μM ATO, or 25 μM ATO for 24 hrs, media was discarded and RNA extracted from the cells using the RNA extraction kit (Ambion, Austin, TX, USA) as previously described [25]. RNA (0.4 μg) was converted into cDNA using the High Capacity cDNA RT-PCR kit (Applied Biosystems, Carlsbad, CA, USA). Quantitative real-time PCR analyses were performed using primers from Integrated DNA Technologies. Amplification reactions were carried out in an ABI Prism Fast 7500 system (Applied Biosystems Inc, Foster City, CA). Gene expression for thymidylate synthase (TS) was performed using the following primer sets from Integrated DNA Technologies: TS forward 5′-GGCCTCGGTGTGCCTTT-3′, reverse 5′-GATGTGCGCAATCATGTACGT-3′. Power SYBR Green PCR Master Mix (Applied Biosystems) was used for the PCR to a final volume of 20 μL. A β-actin primer set (Ambion) was used as an internal control. Cycling conditions were 50°C for 2 minutes and 95°C for 10 mins and followed by 40 cycles at 95°C for 15 sec and 60°C for 1 min. The standard curve method was used to obtain threshold values (C_T_). Triplicate C_T_ values were analyzed in Microsoft Excel using the comparative C_T_ method as described by the manufacturer (Applied Biosystems). Fold differences were calculated as described previously [22] and represent changes normalized to a vehicle reference.

### Statistical analysis

GraphPad Prism 5 (GraphPad Software, San Diego, CA) was used for nonlinear regression statistical analysis. For each concentration, percent inhibition values were calculated and IC_50_ values determined using a four-parameter dose–response (variable slope) equation in GraphPad Prism.

## Results

### HepG2 and LX2 cells are sensitive to the cytotoxic effects of arsenic trioxide

**Figure 1:**
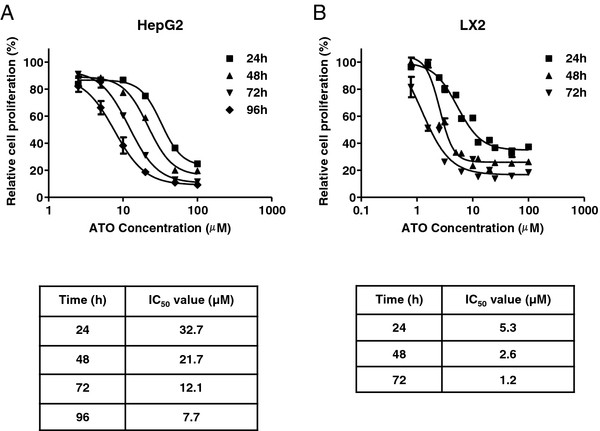
**ATO inhibits HepG2 and LX2 Cell Proliferation.** HepG2 (**A**) and LX2 (**B**) cells were treated with ATO at the indicated concentrations for 24 hrs, 48 hrs, 72 hrs and 96 hrs (HepG2 only) and cell proliferation assessed by MTT assay. For each concentration, percent inhibition values were calculated and data normalized to vehicle control. Relative IC_50_ values shown in the corresponding tables were determined by non-linear regression in GraphPad Prism (n = 3 replicates).

**Figure 2:**
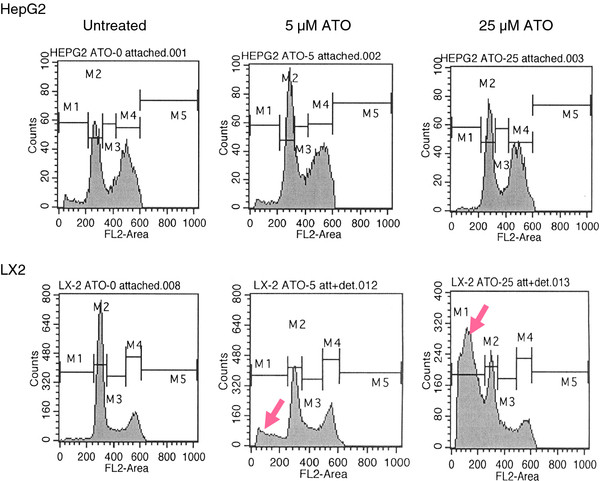
**ATO induces apoptosis of LX2 cells.** HepG2 and LX2 cells were untreated or incubated in the presence of 5 μM or 25 μM ATO for 24 hrs. Apoptotic cells were determined by propidium iodide staining and FACS analysis. The red arrow indicates the sub –G1 peak which represents an early apoptotic cell population. A representative example of three independent experiments is shown.

In order to evaluate the effect of single agent ATO on cellular proliferation, HepG2 and LX2 cells were treated with increasing concentrations of drug for 24 to 96 hr. Dose response curves demonstrate the sensitivity of both cell lines in a time-dependent fashion, with LX2 cells being more sensitive to ATO treatment with a relative IC_50_ of 5.3 μM at 24 hrs of treatment compared to a relative IC_50_ of 32.7 μM for the HepG2 cells at the same incubation time (Figures 1A and 1B). In order to examine the mechanism of ATO-induced cytotoxicity, cell cycle analysis was performed on HepG2 and LX2 cells treated with 5 μM or 25 μM of ATO for 24 hr. Flow cytometry of the LX2 cells demonstrates an early apoptotic population, as represented by the presence of a sub-G1 peak, in cells treated with 5 μM and 25 μM ATO (Figure 2) in comparison to vehicle control. 50 μM ATO treatment is required to induce apoptosis of the HepG2 cells thus confirming the findings of our cytotoxicity assays (data not shown)**.** Taken together, these data indicate that the hepatic stellate cells are more sensitive than the hepatocellular carcinoma cells to ATO-induced cell death.

### Synergistic cytotoxicity of arsenic trioxide in combination with fluorouracil in HepG2 cells

**Figure 3:**
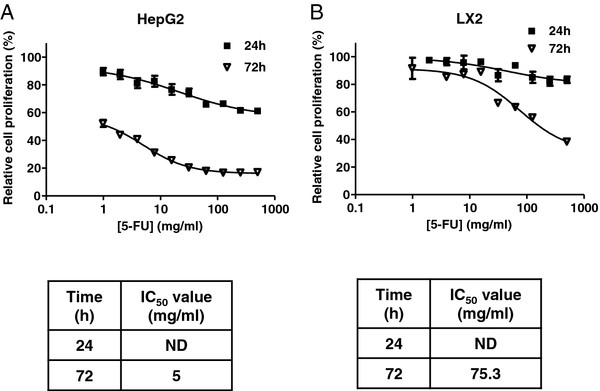
**5-FU inhibits HepG2 and LX2 Cell Proliferation at 72 hrs.** HepG2 (**A**) and LX2 (**B**) cells were treated with 5-FU at the indicated concentrations for 24 and 72 hrs and cell proliferation was assessed by MTT assay. For each concentration, percent inhibition values were calculated and data normalized to vehicle control. IC_50_ values shown in the corresponding tables were determined by non-linear regression in GraphPad Prism (n = 3 replicates).

To reduce variability and facilitate multiple cell line and drug combinations, dose response and drug combination experiments were carried out in 96-well plate format using an automated, high throughput system. First, to determine the degree of cytotoxicity of 5-FU alone on the LX2 and HepG2 cells, both cell lines were treated with increasing concentrations of 5-FU for 24 and 72 hr. Neither cell type experienced significant cytotoxicity following 24 hr of treatment, but cytotoxicity was observed in HepG2 cells treated with 5-FU for 72 hr (IC_50_ of 5 mg/ml at 72 hr) (Figure 3A). In contrast, LX2 cells remain resistant to 5-FU even with high drug concentrations and prolonged exposure (Figure 3B).

**Figure 4:**
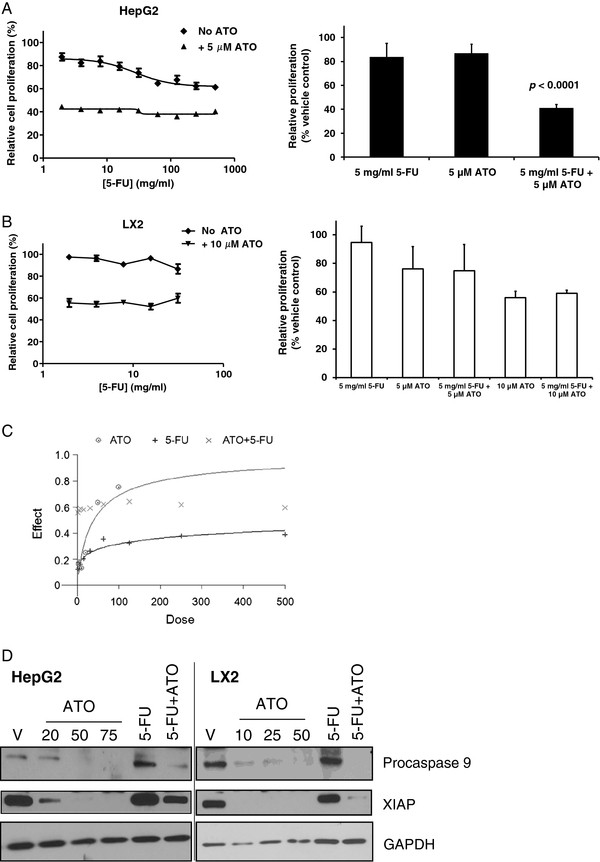
**Combination of ATO with 5-FU in HepG2 and LX2 cell lines.** HepG2 cells (**A**) were treated with increasing concentrations of 5-FU in the absence or presence of ATO (5 μM) for 24 hrs (left panel). The bar graph (right panel) demonstrates relative proliferation values for HepG2 cells when treated with 5 mg/ml 5-FU alone, 5 μM ATO alone and the combination of 5 mg/ml 5-FU and 5 μM ATO (p value < 0.0001). (**B**) LX2 cells were treated with increasing concentrations of 5-FU in the absence or presence of ATO (10 μM) for 24 hrs (left panel). The bar graph (right panel) demonstrates relative proliferation values for LX2 cells when treated with 5 mg/ml 5-FU alone, 5 μM, 10 μM ATO alone and the combination of 5 mg/ml 5-FU + 5 μM or 10 μM ATO. For each concentration, percent inhibition values were calculated and data normalized to vehicle control and represented as n = 3 replicates+/− SEM. (**C**) Dose effect curve for ATO and 5-FU combination in HepG2 cells was generated using Calcusyn software. (**D**) HepG2 treated cell lysates (vehicle, 20 μM, 50 μM or 75 μM ATO, 5 mg/ml 5-FU or 5 μM ATO + 5 mg/ml 5-FU) and LX2 treated cell lysates (vehicle, 10 μM, 20 μM or 50 μM ATO, 5 mg/ml 5-FU or 10 μM ATO + 5 mg/ml 5-FU) assessed for procaspase-9 and XIAP expression by western blot analysis. GAPDH was utilized as a loading control.

To assess potential synergy between 5-FU and ATO, HepG2 and LX2 cells were treated for 24 hrs with increasing concentrations of 5-FU in the absence or presence of a fixed concentration of ATO. At 24 hrs 5-FU has minimal effect on cell proliferation of HepG2 (Figure 4A, left panel) or LX2 cells (Figure 4B, left panel). ATO treatment (5 μM) alone of HepG2 cells for 24 hr also results in minimal cell death, but in combination with 5 mg/ml 5-FU, there is a significant decrease in cell proliferation with the combination treatment (*p* value <0.0001) (Figure 4A, right panel). In contrast, LX2 cells treated with the combination of 5-FU and ATO demonstrated no additional cytotoxicity over that which was seen with ATO treatment alone (Figure 4B, right panel). We have tested ATO at both 5 and 10 μM concentrations for LX2 cells. As the inhibition curve for ATO treatment of LX2 cells at 24 h (Figure 1B) plateaued well above 0% at ~40% it is difficult to summarize such a curve in a single value as there are effects on both potency and efficacy. Hence, we chose to test both the calculated IC_50_ concentration (relative IC_50_ value of 5 μM) and the concentration that reduced proliferation by 50% (absolute IC_50_ value of 10 μM). LX2 cells treated with the combination of 5-FU and ATO at either concentration (10 μM data shown in Figure 4A left panel and both 5 and 10 μM shown in Figure 4A, right panel) demonstrated no additional cytotoxicity over that which was seen with ATO treatments alone.

To further characterize the interaction between 5-FU and ATO, the dose-effect curves were analyzed using Calcusyn software. The combination index (CI) for the interaction between ATO (5 μM) and 5-FU (increasing dose) in HepG2 cells was calculated to be 0.098-0.159 (Figure 4C), which is indicative of strong synergism in this cell model. To assess the effect of ATO and 5-FU treatment on apoptosis, expression of procaspase-9, the inactive form of the apoptotic effector caspase-9, and X-linked inhibitor of apoptosis protein (XIAP), the most potent mammalian caspase-inhibitor, was examined in HepG2 and LX2 cell lysates. Data in Figure 4D show that procaspase 9 and XIAP decrease in cells undergoing death at lower concentration with ATO in LX2 cells compared to HepG2 cells consistent with enhanced potency of ATO in LX2 cells. Procaspase-9 expression was preserved in HepG2 cells treated with 5 mg/ml 5-FU or 20 μM ATO alone. 5-FU as a single agent at 5 mg/ml did not cause any decrease in procaspase 9 or XIAP in either cell lines. However, expression was abolished when cells were treated with 5 mg/ml 5-FU and 5 μM ATO in combination (Figure 4D), suggesting enhanced activation of apoptosis by this combination in HepG2.

### ATO inhibits thymidylate synthase expression in a dose dependent fashion

**Figure 5:**
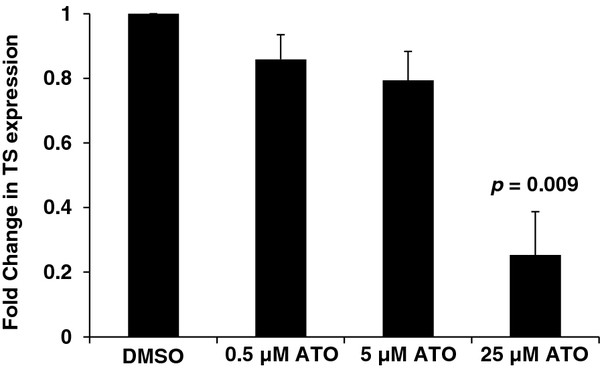
**ATO inhibits thymidylate synthase expression in a dose dependent fashion.** HepG2 cells were treated for 24 hrs with vehicle, 0.5 μM, 5 μM or 25 μM ATO. Thymidylate synthase mRNA expression was assessed by qRT-PCR and normalized to actin levels. Each sample was run in triplicate. A representative example of four independent experiments is shown. Bar graph demonstrates fold change relative to vehicle control +/− SEM.

An earlier study in colorectal adenocarcinoma cells has shown that a combination of ATO and 5-FU results in enhanced cytotoxicity due in part to ATO-induced inhibition of thymidylate synthase (TS) [13]. To evaluate the effect of ATO on TS expression in HepG2 cells, cells were treated with DMSO control, 0.5 μM ATO, 5 μM ATO or 25 μM ATO for 24 hours. Quantitative real-time PCR analysis demonstrated that there was a dose dependent reduction in TS expression (Figure 5). ATO-induced modulation of TS levels may, in part, account for the enhanced apoptosis with the 5-FU combination as seen in Figure 4.

### ATO treatment of HepG2 cells results in sustained activation of MAPK and JNK

**Figure 6:**
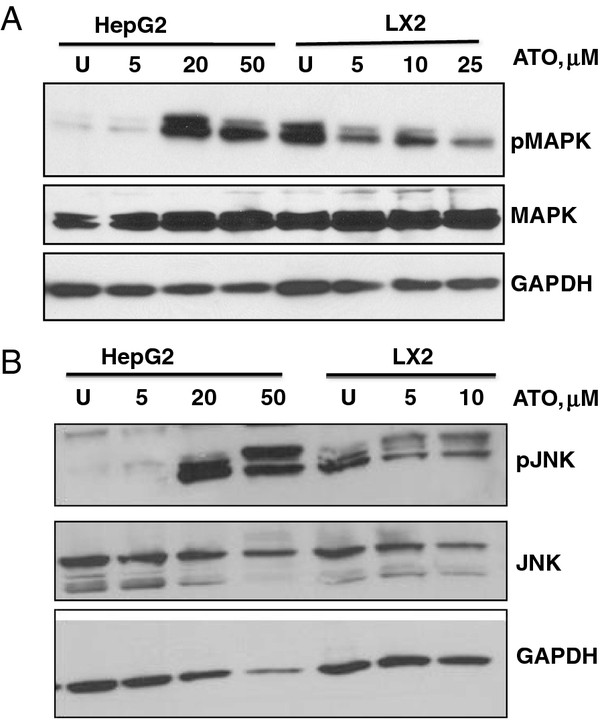
**ATO treatment of HepG2 cells results in sustained activation of MAPK and JNK.** HepG2 and LX2 cells were treated with vehicle or 5–50 μM ATO as indicated for 24 hrs. Levels of phosphorylated and total MAPK (**A**) and JNK (**B**) were assessed by western blot analysis. GAPDH was utilized as a loading control.

In order to query the mechanism of HepG2 resistance to ATO, we examined the status of two cell survival pathways, the mitogen-activated protein kinases, ERK1 and ERK2, and Janus-associated kinase (JNK), in ATO-treated cells. Treatment of HepG2 cells at concentrations of ATO that induce cell death at 24 hr resulted in robust activation of the mitogen-activated protein kinases, (pERK1 and pERK2, Figure 6A) and (pJNK, Figure 6B) which was not observed in ATO-treated LX2 cells (pERK1 and pERK2, Figure 6A) and (pJNK, Figure 6B).

### Combination treatment with arsenic trioxide and sorafenib shows a trend towards increased cytotoxicity and attenuates p-MAPK

**Figure 7:**
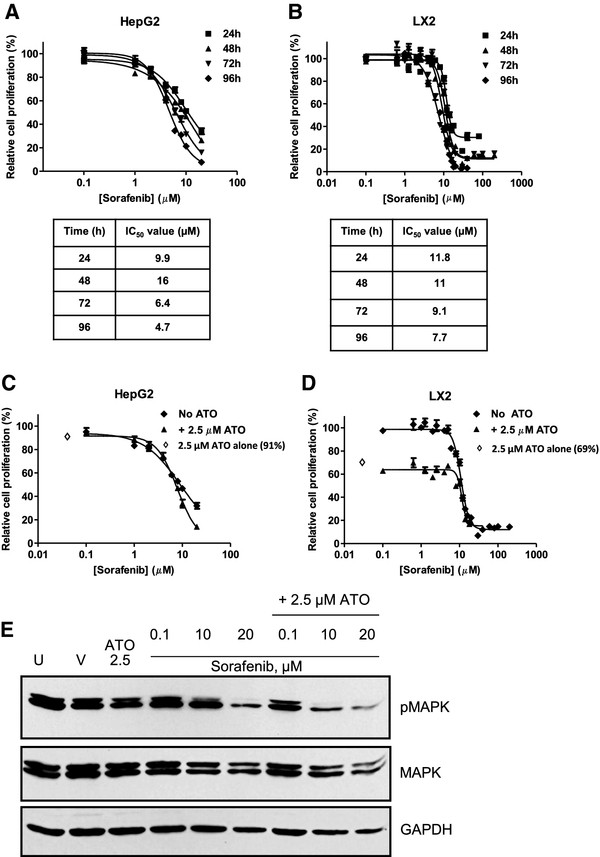
**Treatment of HepG2 cell lines with ATO and Sorafenib results in enhanced cytotoxicity and inhibits MAPK activation.** HepG2 (**A**) and LX2 (**B**) cells were treated with Sorafenib at the indicated concentrations for 24 hr, 48 hr, 72 hr and 96 hr and cell proliferation assessed by MTT assay. For each concentration, percent inhibition values were calculated and data normalized to vehicle control. IC_50_ values shown in the corresponding tables were determined by non-linear regression in GraphPad Prism. HepG2 (**C**) and LX2 (**D**) cells were treated with increasing concentrations of Sorafenib in the absence or presence of ATO (2.5 μM) for 48 h. Inhibition values for ATO alone (2.5 μM) are shown (◊). (**E**). HepG2 cells were untreated or treated with vehicle, 2.5 μM ATO or 0.1–20 μM sorafenib +/− 2.5 μM ATO as indicated for 24 hrs. Phosphorylated and total MAPK levels were assessed by western blot analysis. GAPDH was utilized as a loading control.

The induction of a delayed and sustained survival signal as seen by pERK and pJNK activation in HepG2 cells (Figure 6) may potentially contribute to an ATO resistance mechanism. Based on observations in other systems that combination treatment with ERK or JNK inhibitors restores chemosensitivity, we hypothesized that treatment with ATO and the multikinase inhibitor, sorafenib, would be more efficacious than either agent alone. In order to evaluate the effect of sorafenib on cellular proliferation, HepG2 and LX2 cells were treated with increasing concentrations of drug for 24 to 96 hr. Sorafenib has significant cytotoxic effects on both HepG2 and LX2 cells in a dose and time dependent fashion (24 hr; HepG2 IC_50_ 9.9 μM, LX2 IC_50_ 11.8 μM; Figure 7A and 7B). To assess synergism of the two agents, HepG2 and LX2 cells were treated with 2.5 μM ATO in the presence of increasing concentrations of sorafenib for 24 hr (Figure 7C and 7D). At 24 hr there was no significant difference in IC_50_ values for the combination in comparison to sorafenib alone in both the HepG2 and LX2 cells. However at the later time points of 48 and 72 hr, the percent of proliferating HepG2 cells was less for 20 μM sorafenib + 2.5 μM ATO than for 20 μM sorafenib alone (at 48 hr 13.7% versus 31%, p < 0.05; data not shown). The increased cytotoxicity was accompanied by a decrease in phospho-MAPK levels in HepG2 cells treated with ATO and sorafenib in comparison to ATO alone (Figure 7E). ATO treatment of LX2 cells results in potent cytotoxicity and the addition of sorafenib resulted in no further additive effect**.**

### Discussion and conclusions

ATO has demonstrated promising single agent activity in both *in vitro* and *in vivo* models of HCC, but as a single agent, has had limited activity in HCC clinical trials. However, its ability to induce apoptosis supported the objective of this study, which was to identify agents that demonstrate synergy with ATO in HCC. We were interested in studying the effect of these combinations on the tumor epithelial cells and stromal cells as a growing body of literature highlights the cross-talk between malignant hepatocytes and the surrounding peri-tumoral stroma as a key modulator of hepatocarcinogenesis and chemoresistance [1].

One of the major strengths of our work is the simultaneous assessment of cytotoxicity on a malignant hepatic cell line, HepG2 cells, and a hepatic stellate cell line, LX2 cells. In healthy livers, quiescent hepatic stellate cells (Q-HSCs) reside in the sinusoidal space, store retinoids, and produce molecules that are trophic for neighboring hepatocytes. In the setting of chronic liver injury, quiescent HSCs are transformed to myofibroblastic hepatic stellate cells (MF-HSCs), producing the extensive extracellular matrix that accumulates with liver fibrosis [26,27]. As liver fibrosis is the most important determinant of risk for the development of HCC, MF-HSCs are believed to play an important role in driving tumor formation. MF-HSCs infiltrate the stroma of HCCs, localizing around tumor sinusoids, fibrous septa and capsules where they elaborate a variety of cytokines, chemokines and growth factors [28,29]. Conditioned medium derived from MF-HSCs has been shown to significantly induce the proliferation and migration of malignant hepatocytes *in vitro*[2]. The importance of MF-HSCs in hepatocarcinogenesis was recently confirmed by *in vivo* co-transplantation experiments in which Ras-transformed hepatocytes were co-injected with MF-HSCs. Co-transplantation strongly accelerated tumor growth, resulting in a 3-fold increase in tumor volumes as compared with those generated by Ras-transformed hepatocytes alone [5]. Taken together, these data suggest that in the further development of therapeutics for HCC, the effect of compounds on both the epithelial and the stromal compartment should be assessed.

Although sorafenib is FDA-approved for patients with advanced HCC based on a modest survival benefit, there is considerable interest in identifying more efficacious drugs and combinations. We observed that both HepG2 cells and LX2 cells are sensitive to ATO and that treatment combinations with either sorafenib or 5-FU lead to enhanced cytotoxicity. Importantly, while HepG2 cells are relatively resistant to 5-FU monotherapy, when combined with ATO, synergy is observed as previously demonstrated in 5-FU resistant colorectal cancer cells [13,30]. Our data indicates that the enhanced apoptosis associated with the combination may, in part, be mediated by an ATO-induced, dose-depenedent decrease in thymidylate synthase. Subtle changes in the level of this enzyme may affect sensitivity to 5-FU, but certainly, other mechanisms may also place a role in the synergy of ATO and 5-FU. Similarly, the addition of ATO to sorafenib in HepG2 cells results in greater cytotoxicity than either agent alone likely due to potent inhibition of MAPK activation. Taken together these results suggest that the addition of ATO leads to the enhanced cytotoxicity of 5-FU and sorafenib.

Our data demonstrate that ATO induces apoptosis in both the HepG2 and LX2 cell lines in a caspase dependent fashion. These findings confirm those of other groups, which have demonstrated that ATO induces apoptosis of multiple solid tumor cell types [31-34]. Furthermore, the concentrations of ATO required for 50% cytotoxicity in our in vitro cell-based proliferation assays (5 μM) are comparable to peak levels measured in the plasma (5.5–7.3 μM) of patients undergoing standard ATO treatment for acute promyelocytic leukemia suggesting that these doses of ATO can be therapeutically achieved [35]. However, it remains a possibility that higher doses of ATO will be required for optimal cytotoxicity in the context of HCC.

Compared to the HepG2 cells, the LX2 cells are highly sensitive to the cytotoxic effects of single agent ATO. In the HepG2 cells, addition of 5-FU to ATO causes a leftward shift in the cytotoxicity curve, revealing a potential synergy, and importantly, the combination appears to target both the epithelial and stromal compartments. The synergy of this combination was suggested to be due to decrease of thymidylate synthase levels induced by ATO. Indeed, in a phase I clinical trial of 12 patients with metastatic, refractory colorectal cancer treated with the combination of ATO and 5-FU [36], correlative studies demonstrated that ATO caused a significant decrease in thymidylate synthase mRNA levels in peripheral blood mononuclear cells. Similar findings were noted in post-treatment tumor biopsies. With the caveat of small patient numbers, there appeared to be a correlation between degree of TS reduction and median progression free survival and overall survival. Based on these data the authors hypothesized that ATO-induced downregulation of TS results in resensitization of colorectal cancer to 5-FU [13,37].

In order to query the mechanism of relative HepG2 resistance to ATO, we examined the status of cell survival pathways in ATO-treated cells. Paradoxically, ATO treatment of HepG2 cells at concentrations that induce cell death resulted in robust activation of the MAPK and JNK pathways. Activation of these pathways was not observed in ATO-treated LX2 cells**.** Similar findings have been described in multiple other cell types treated with various pro-oxidants such as cisplatin [38] and thymoquinone [39]. The accumulation of reactive oxygen species results in activation of survival and proliferative signals that mediate tumorigenicity and chemoresistance [39-41]. This induction of a delayed and sustained survival signal by the HepG2 cells potentially contributes to an ATO resistance mechanism. Similar to observations in other tumor types where combination treatment with ERK or JNK inhibitors restores chemosensitivity [39], we demonstrate enhanced cytotoxicity of the HepG2 cells with the ATO/sorafenib combination in comparison to ATO alone. While studies are needed to further clarify the exact mechanism of cytoxicity of the ATO combinations, these data provide strong evidence that ATO/5-FU and ATO/sorafenib demonstrate enhanced cytoxicity of the epithelial and stromal compartment. These are rational combinations to further explore for the treatment of patients with advanced HCC.

## Abbreviations

ATO: Arsenic trioxide; 5-FU: 5-fluorouracil; HCC: Hepatocellular carcinoma; EMT: Epithelial to mesenchymal transition; CI: Combination index; PI: Propidium iodide; TS: Thymidylate synthase; JNK: Janus associated kinase; ERK: Mitogen activated protein kinase; Q-HSCs: Quiescent hepatic stellate cells; MF-HSCs: Myofibroblastic hepatic stellate cells.

## Competing interests

The author(s) declare that they have no competing interests.

## Authors' contribution

FR conceived and designed experiments, analyzed and interpreted data, drafted/revised the manuscript; GS performed data collection, analyzed data; KPW conceived and designed experiments, coordinated data collection, performed data analysis and interpretation, and revised the manuscript; ZT performed data collection, analyzed data; JLA performed data collection, analyzed data; HKL conceived and designed experiments, interpreted data and revised the manuscript; AMD conceived and designed experiments, interpreted data and revised the manuscript; MAM conceived and designed experiments, interpreted data, drafted and revised the manuscript; GRD conceived and designed experiments, coordinated data collection, analyzed and interpreted data, drafted and revised the manuscript. All authors read and approved the submitted version of the manuscript.

## Authors' information

Michael A Morse, Gayathri R Devi are co-senior authors.

## Pre-publication history

The pre-publication history for this paper can be accessed here:

http://www.biomedcentral.com/1471-2407/12/402/prepub
